# Vertical Transmission Selects for Reduced Virulence in a Plant Virus and for Increased Resistance in the Host

**DOI:** 10.1371/journal.ppat.1004293

**Published:** 2014-07-31

**Authors:** Israel Pagán, Nuria Montes, Michael G. Milgroom, Fernando García-Arenal

**Affiliations:** 1 Centro de Biotecnología y Genómica de Plantas (UPM-INIA) and Departamento de Biotecnología, Campus Montegancedo, Universidad Politécnica de Madrid. Pozuelo de Alarcón, Madrid, Spain; 2 Department of Plant Pathology and Plant-Microbe Biology, Cornell University, Ithaca, New York, United States of America; University of California, Davis Genome Center, United States of America

## Abstract

For the last three decades, evolutionary biologists have sought to understand which factors modulate the evolution of parasite virulence. Although theory has identified several of these modulators, their effect has seldom been analysed experimentally. We investigated the role of two such major factors—the mode of transmission, and host adaptation in response to parasite evolution—in the evolution of virulence of the plant virus *Cucumber mosaic virus* (CMV) in its natural host *Arabidopsis thaliana*. To do so, we serially passaged three CMV strains under strict vertical and strict horizontal transmission, alternating both modes of transmission. We quantified seed (vertical) transmission rate, virus accumulation, effect on plant growth and virulence of evolved and non-evolved viruses in the original plants and in plants derived after five passages of vertical transmission. Our results indicated that vertical passaging led to adaptation of the virus to greater vertical transmission, which was associated with reductions of virus accumulation and virulence. On the other hand, horizontal serial passages did not significantly modify virus accumulation and virulence. The observed increases in CMV seed transmission, and reductions in virus accumulation and virulence in vertically passaged viruses were due also to reciprocal host adaptation during vertical passages, which additionally reduced virulence and multiplication of vertically passaged viruses. This result is consistent with plant-virus co-evolution. Host adaptation to vertically passaged viruses was traded-off against reduced resistance to the non-evolved viruses. Thus, we provide evidence of the key role that the interplay between mode of transmission and host-parasite co-evolution has in determining the evolution of virulence.

## Author Summary

Virulence is a key property of parasites, and is linked to the emergence of new diseases and to the reduction of ecosystem biodiversity. Consequently, scientists have devoted a great effort to build theoretical models that predict which factors may modulate virulence evolution. However, whether (and how) these factors affect virulence evolution has been seldom analysed experimentally. Using the plant virus *Cucumber mosaic virus* (CMV) and its natural host *Arabidopsis thaliana*, we studied the role of two such factors: the mode of transmission, and host adaptation in response to parasite evolution. We serially passaged CMV under strict vertical and strict horizontal transmission, and a combination of both. Subsequently, we analysed differences in CMV seed (vertical) transmission rate, accumulation and virulence between evolved and non-evolved viruses. We also compared whether these differences varied in original plants and in plants evolved during vertical passaging. Vertical passaging increased CMV seed transmission, and reduced accumulation and virulence, while horizontal passaging had no effect. Changes during vertical passaging were determined also by reciprocal host adaptation, which additionally reduced virulence and accumulation of vertically transmitted viruses. Hence, we provide evidence that the interplay between the transmission mode and host-parasite co-evolution is central in determining virulence evolution.

## Introduction

Understanding which factors determine the evolution of virulence–the negative effect of parasites on host fitness [1], [2] –and how they act, is a long-standing goal in evolutionary biology, and central for the control of infectious diseases [3], [4]. Indeed, changes in virulence have been associated with the effects of parasites on host population dynamics [1], [5], the reduction of ecosystem biodiversity [6], [7], and the emergence and re-emergence of infectious diseases [8],[9]. In the last three decades considerable effort has been devoted to developing theoretical models that predict conditions favouring the increase or decrease of parasite virulence. Most models are based on the hypothesis that the level of virulence is determined by trade-offs between the within-host and between-host components of the parasite's fitness; this is known as the trade-off hypothesis [2], [10]–[12]. According to the trade-off hypothesis the level of virulence is generally, but not always [13], [14], a consequence of optimizing the within-host multiplication and between-host transmission components of parasite fitness [10], [13]–[16]. Two key assumptions underlie the trade-off hypothesis. First, virulence is positively correlated with parasite multiplication within the infected host; and second, greater parasite load in an infected host increases the probability of transmission to a susceptible, uninfected host. A trade-off occurs because higher virulence may also increase host mortality, reducing the infectious period and the probability of transmission.

Experimental analyses have shown that multiplication rates and/or transmission rates are positively correlated with virulence for most parasites of humans, animals and plants when transmitted horizontally, i.e., between host individuals that are not parent and offspring [2], [17]–[19]; [ see 20], [ 21 for exceptions]. However, a wide range of human, animal and plant parasites that cause severe diseases, are vertically transmitted, i.e., from parent to offspring, or are transmitted both horizontally and vertically. Evolution of virulence in these parasites may challenge the trade-off hypothesis and derived models, since the presence of an alternative mode of transmission may reduce the relative importance of the trade-off between horizontal transmission and virulence. For instance, under strict vertical transmission, virulence should be negatively correlated with transmission rate [2], [13], [14], [22]: The fitness of vertically transmitted parasites is highly dependent on host reproductive potential, as hosts need to reproduce for the parasite to infect new individuals. Since virulence, by definition, reduces host fitness, vertically transmitted parasites should evolve towards lower virulence to maximize their own fitness [10], [13], [14], [23]–[25]. Accordingly, the ‘continuum hypothesis’ proposes that the optimum virulence in parasites transmitted both vertically and horizontally will vary along a continuum depending on the relative weight of each transmission mode on the parasite's fitness: parasites mostly vertically transmitted will tend towards lower virulence and parasites mostly horizontally transmitted will tend towards higher virulence [14], [16], [22].

Despite the abundance of theory, and the numerous examples of important parasites with vertical or mixed modes of transmission, the largest fraction of experimental analyses of the effect of transmission mode on the evolution of virulence has been done under strict horizontal transmission. Studies of parasite evolution under vertical transmission have mostly reported a negative correlation between virulence and rate of vertical transmission, supporting the ‘continuum hypothesis’ [26]–[33]. However, this might not be a universal trend. For instance, in some of the few plant-parasite interactions studied, virulence was not negatively correlated with vertical transmission [34], [35]. Therefore, understanding the relationship between mode of transmission and virulence evolution requires further analysis and is in need of more experimental data from a larger variety of host-parasite systems [36].

Most theoretical and experimental analyses of virulence evolution overlook the important fact that virulence and transmissibility are not just parasite traits [8]. Rather, virulence and transmissibility are the result of the parasite's interaction with the host, and thus are potentially subjected to host-parasite co-evolution [8], [37], [38]. For instance, reduced virulence might result from selection on the parasite to increase the efficiency of its vertical transmission, or from reciprocal selection on the host to reduce the damage caused by the parasite during vertical transmission [3], [39]. Selection on the host will thus result in higher tolerance, where tolerance is defined as the host's ability to reduce the effect of infection on its fitness [8], [40]. For example, tolerance of *Arabidopsis thaliana* plants to virus infection is achieved through developmental reprogramming, so that resources are reallocated from the vegetative growth to reproduction [41]. Demonstrating co-evolution presents the daunting challenge of demonstrating reciprocal effects of both host and pathogen [37]. Thus, empirical evidence for the co-evolution of host- and parasite-related components of virulence is limited [8], [37].

Here we analyse experimentally the role of the mode of transmission, vertical or horizontal, on the evolution of virulence, while also accounting for the effect of host-parasite co-evolution in virulence-related traits. For this, we used the plant-virus system *Arabidopsis thaliana* L. Heynh. (Brassicaceae) - *Cucumber mosaic virus* (CMV, *Bromoviridae*). *A. thaliana* (from here on, *Arabidopsis*) has been developed as model organism for molecular and genetic analyses of a wide range of plant traits, and also is increasingly used in analyses of host-parasite co-evolution [e.g. 42–48]. The short life cycle [49], [50] of *Arabidopsis* facilitates performing serial passage experiments of vertical transmission, and the large numbers of seeds produced by a plant [49], [51] ensures the maintenance of parasite lineages between host generations even at low rates of vertical transmission. CMV is a generalist parasite with the broadest host range known for a plant virus, and its genomic structure, replication, gene expression and pathogenicity have been analysed extensively [reviewed in 52], [ 53]. CMV isolates are highly diverse and they have been classified into two subgroups (subgroup I and subgroup II) based on the nucleotide sequence similarity of their genomic RNAs. CMV is horizontally transmitted by more than 70 species of aphids in a non-persistent manner, and vertically through seeds, with rates that vary depending on CMV and plant genotypes [53]. In *Arabidopsis*, the efficiency of CMV seed transmission ranges between 2 and 8% [unpublished data].

In this work, we serially passaged three CMV strains in one *Arabidopsis* genotype for five generations under three transmission modes: strict vertical transmission, strict horizontal transmission, and alternation of vertical and horizontal transmission. We monitored the vertical transmission rate through seeds, virus accumulation and virulence after each passage. After the last passage, we compared virus accumulation and virulence of the evolved and the non-evolved virus lineages, both in the original plant stock, and in the progeny of plants derived from the fifth passage of vertical transmission. Results indicate that vertical passaging led to adaptation of the virus to greater vertical transmission, which was associated with reductions of virus accumulation and virulence. Increases in seed transmission and reductions in virus accumulation and virulence were determined also by reciprocal host adaptation during vertical passages, which additionally reduced virulence and multiplication of vertically evolved viruses. Host adaptation to vertically passaged viruses was traded-off against reduced resistance to the non-evolved viruses.

## Results

### Evolution of seed transmission rate, virus accumulation and virulence during vertical passages

**Figure 1:**
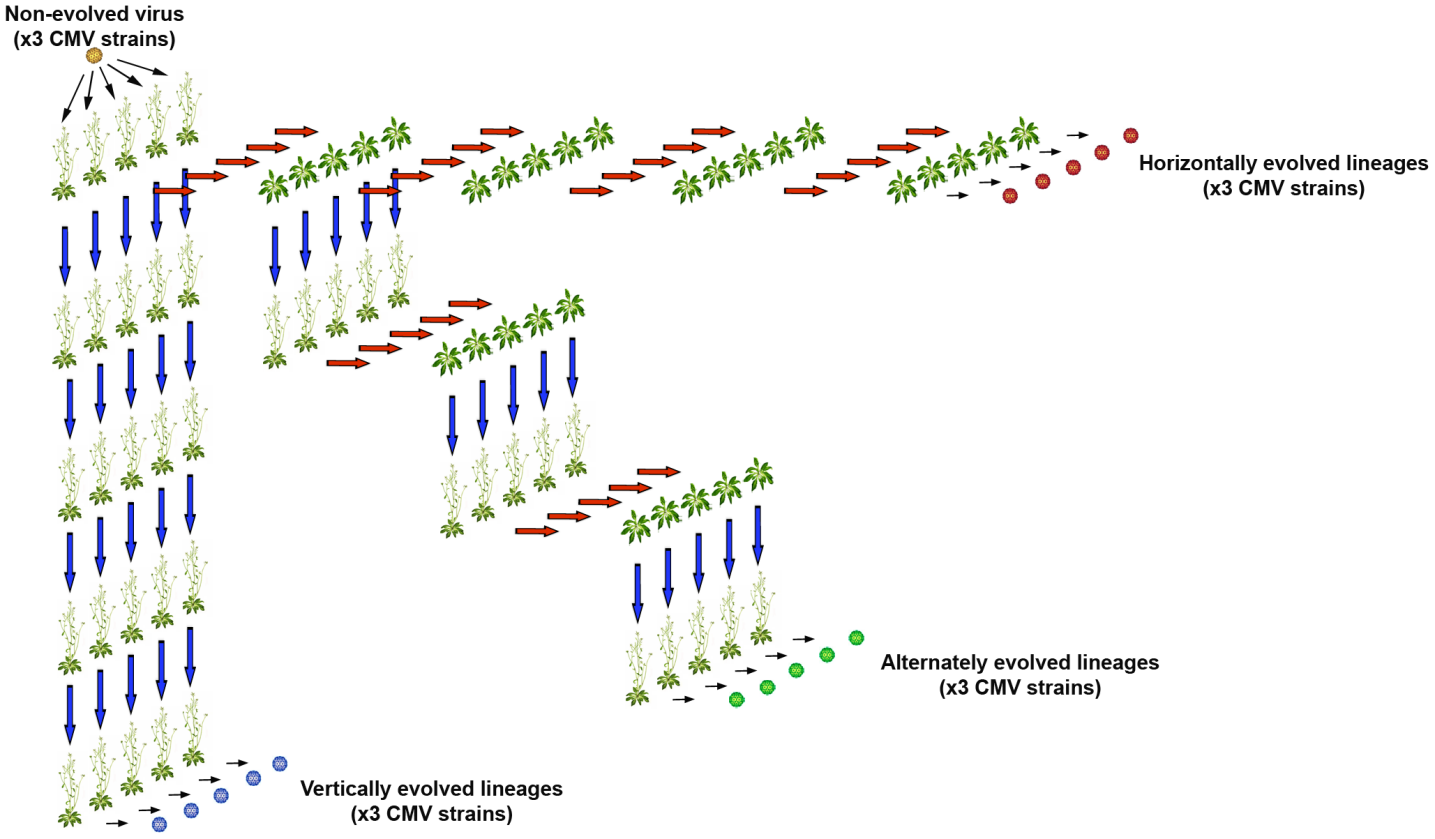
Experimental design. Three CMV strains were inoculated in five Cen-1 *Arabidopsis* plants each. Each of these 15 plants represented the origin of a lineage of vertically transmitted viruses indicated by blue arrows (left column), another lineage of horizontally transmitted viruses indicated by red arrows (upper row), and a third group of lineages in which vertical and horizontal transmission modes were alternated (diagonal).

**Figure 2:**
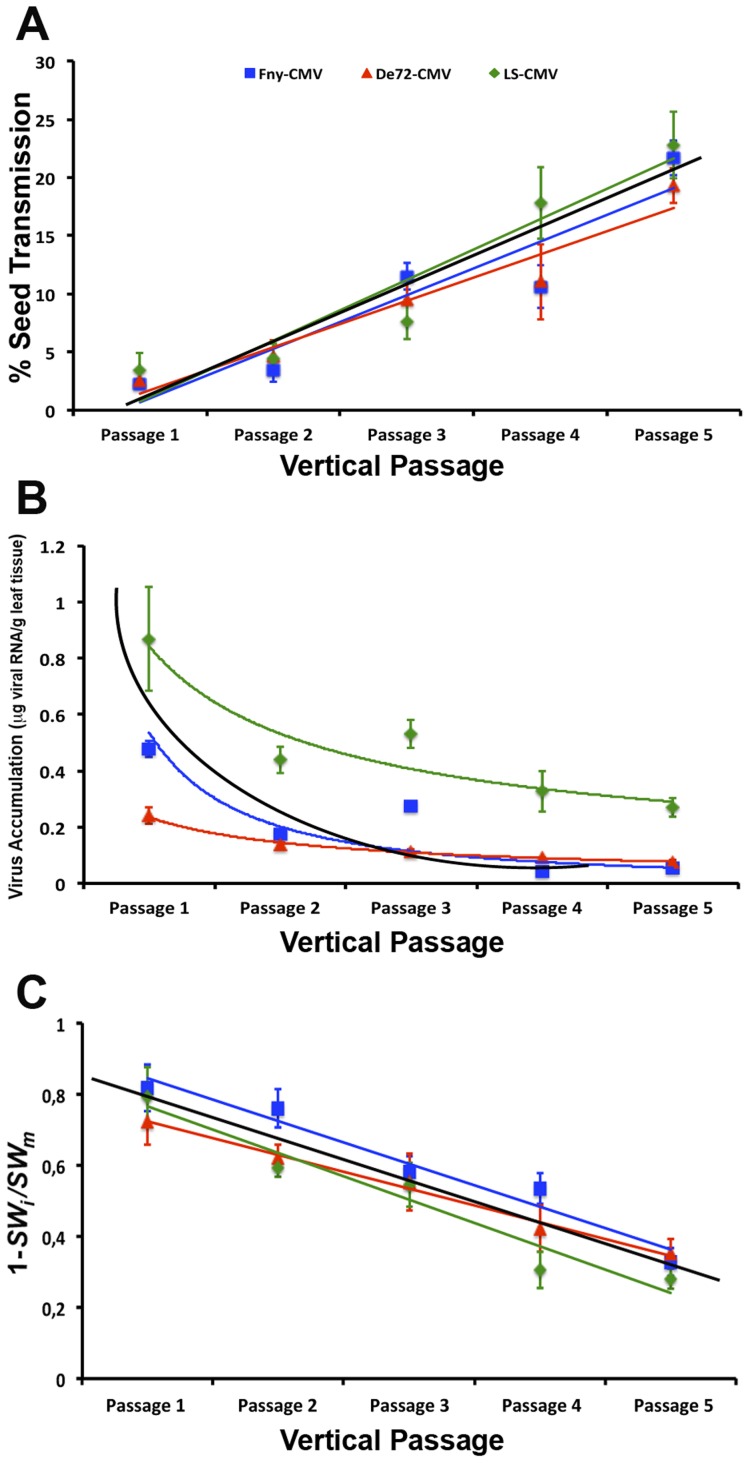
Evolution of CMV seed transmission, virus accumulation and virulence across passages of vertical transmission. Seed transmission rate (**A**) was estimated from the number of infected seedlings out of 100 seeds per plant. Virus accumulation (**B**) was measured as µg of viral RNA per g of fresh leaf tissue. Virulence (**C**) is represented as one minus the ratio of seed weight in infected to mock-inoculated plants: 1−(*SW*_i_ */SW*_m_). Data are plotted as mean±standard error of the five independent lineages in each passage for Fny-CMV (blue squares), De72-CMV (red triangles) and LS-CMV (green diamonds). The black line in each panel represents the fitted regression line for lineages of all three strains combined.

We studied the evolution of three strains of CMV; Fny-CMV (a well-characterized strain isolated in New York State, belonging to subgroup I of CMV strains), De72-CMV (isolated from a species in the Brassicaceae in Central Spain and also belonging to subgroup I), and of LS-CMV (a well-characterized strain isolated in New York State, belonging to subgroup II) [53] in *Arabidopsis* under strict vertical, strict horizontal or alternated vertical and horizontal transmission (Figure 1, and see Material and Methods for experimental details). The vertical transmission rate, estimated as the percentage of CMV-infected seeds; virus accumulation, quantified as µg of viral RNA per gr of fresh plant tissue; and virulence, measured as the effect of infection on fecundity, estimated from total seed weight, 1−(*SW_i_/SW_m_*) (*i* and *m* denote infected and mock plants, respectively), were determined in each of five passages of vertical transmission (Figure 2, Table 1). CMV vertical transmission rate, virus accumulation and virulence in each passage were quantified in the same plants in which the virus was passaged. For each of the three virus strains, the five replicate lineages yielded similar values for seed transmission rate (*F_4,25_*≤2.19; *P*≥0.120), virus accumulation (*F_4,25_*≤1.51; *P*≥0.248), and virulence (*F_2,72_*≤1.50; *P*≥0.251). Thus, lineage was not considered as a factor for further analyses.

**Table 1:** Estimates of virus accumulation, virulence and seed transmission rate in vertically infected Cen-1 plants derived from the fifth vertical passage.

Strain	Virus Accumulation^1^	Virulence^2^:1−(*SW_i_/SW_m_*)	% Seed Transmission^3^
Fny-CMV	0.06±0.00	0.33±0.04	21.67±1.48
LS-CMV	0.27±0.03	0.28±0.03	22.80±2.87
De72-CMV	0.08±0.01	0.35±0.04	19.30±1.54

1 Accumulation of virus RNA (µg/g fresh weight) estimated for 1∶1 mix of inoculated and systemically infected leaves.

2 Effect of CMV infection on seed weight (*SW*) estimated as 1−(*SW_i_*/*SW_m_*), where *i* and *m* denote infected and mock-inoculated plants, respectively.

3 Seed transmission rate was estimated as the number of infected seedlings out of 100 seeds.

Values are mean±standard error of the 5 lineages in evolved viruses and 10 replicates in non-evolved strains.

Seed transmission rate increased as the number of vertical passages increased, either when the three CMV strains were analysed together (*F_4,72_* = 48.72; *P*<1×10^−5^), or independently (*F_4,25_*≥12.79; *P*≤1×10^−5^). Indeed, a significant positive linear correlation between seed transmission rate and number of vertical passage was found either when the three strains were considered together (*r* = 0.84; *P*<1×10^−5^) or individually (*r*≥0.83; *P*<1×10^−5^). Slopes and intercepts of these regression lines did not differ significantly between CMV strains (*F_2,72_*≤1.03; *P*≥0.363), indicating that seed transmission increased at the same rate for the three viral strains (Figure 2a, Table 1).

Virus accumulation decreased as the number of vertical transmission passages increased, either considering all strains together (*F_4,72_* = 8.16; *P*<1×10^−4^) or separately (*F_4,25_*≥6.17; *P*≤0.002) (Figure 2b, Table 1). A negative logarithmic correlation between virus accumulation and vertical passage was found considering all strains together (*r* = −0.58; *P*<1×10^−5^) and independently (*r*≥−0.69; *P*≤1×10^−4^). The slope and intercept of the De72-CMV regression were different from those of the Fny-CMV and LS-CMV regressions, so that virus accumulation decreased more slowly in the former than in the latter two strains (*F_2,72_*≥9.41; *P*≤0.010) (Figure 2b).

Virulence decreased as the number of passages increased for all viral strains together and individually (*F_4,72_*≥6.53; *P*≤0.002) (Figure 2c, Table 1). A significant negative linear correlation between virulence and vertical passage was observed, either for all strains together (*r* = −0.80; *P*<1×10^−5^) or separately (*r≤*−0.78; *P*<1×10^−5^). Slopes and intercepts of the regression lines for each viral strain were similar (*F_2,72_*≤1.23; *P*≥0.298).

**Figure 3:**
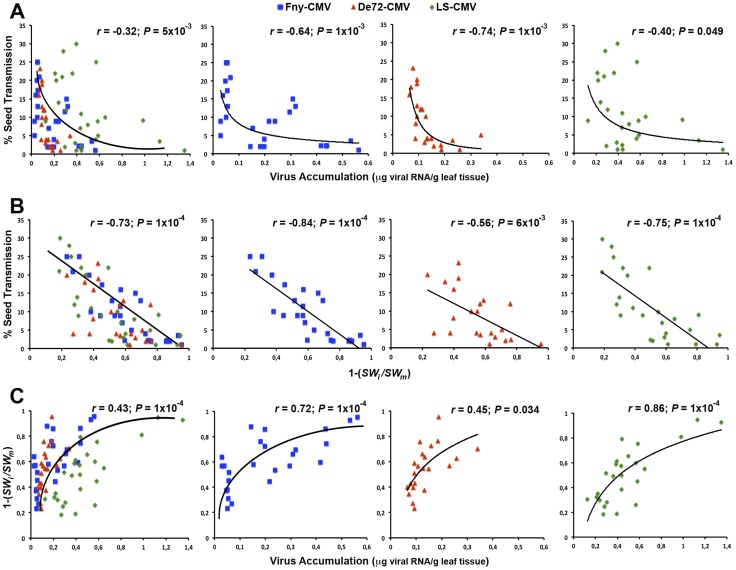
Bivariate analyses between seed transmission, virus accumulation and virulence across passages of vertical transmission. Significant regressions of seed transmission rate on virus accumulation (**A**), seed transmission rate on virulence (one minus the ratio of seed weight in infected to mock-inoculated plants, 1−[*SW_i_*/*SW_m_*]) (**B**), and virulence on virus accumulation (**C**), for each virus strain: Fny-CMV (blue squares), De72-CMV (red triangle) and LS-CMV (green diamonds). Note the different scales depending on the trait and the virus strain.

The analyses above strongly suggest that increases in seed transmission rate might be accompanied by reductions in virus accumulation and virulence. To explore this relationship, we analysed the association between these traits during the serial vertical transmission passages considering all strains together and independently (Figure 3). Regression analyses indicated that the seed transmission rate was negatively (exponentially) correlated with virus accumulation for all strains together (*r* = −0.32; *P* = 0.005) and separately (*r*≤−0.40; *P*≤0.049) (Figure 3a). In addition, seed transmission rate was also negatively (linearly) correlated with virulence in all CMV strains (*r*≤−0.56; *P*≤0.006) (Figure 3b). Finally, virus accumulation and virulence were always positively (exponentially) correlated (*r*≥0.45; *P*≤0.034) (Figure 3c). Overall, these results indicate that the increase of seed transmission rate through vertical passages is associated with a reduction of virus accumulation and virulence in *Arabidopsis*. These changes might be due to virus evolution and/or host evolution. In the next sections we address these possibilities.

### Comparison of virus accumulation and virulence of evolved and non-evolved CMV strains in ‘original’ stock plants

We further analysed how the mode of transmission affected CMV accumulation, and the effect of infection on plant growth and fitness. To do so, virus lineages evolved under vertical, horizontal and alternated transmission (from here on, referred to as vertically evolved, horizontally evolved and alternately evolved viruses), as well as the initial non-evolved strains, were inoculated in plants from the ‘original’ seed stock. In these plants, we determined the effect of virus infection on plant vegetative and reproductive growth, through the weight of the rosettes (*RW*) and inflorescences (*IW*), respectively. We previously reported that tolerance of *Arabidopsis* to CMV is attained by reallocating resources from vegetative to reproductive structures [41]. For these analyses, we used the *RW* and *IW* ratios (*Trait_i_*/*Trait_m_*, where *i* and *m* denote infected and mock-inoculated plants, respectively). We also quantified the effect on *SW* (virulence) and virus accumulation as described above. Vertical transmission rate in plants from the ‘original’ seed stock was quantified only for vertically evolved viruses. The five virus lineages for each combination of CMV strain and mode of transmission did not differ for any of the parameters estimated (*F*≤2.27; *P*≥0.077) (Table S1). Thus, lineage was not considered as a factor for further analyses. Indeed, GLM analyses nesting lineage in mode of transmission did not change our results (not shown).

Virus accumulation differed between evolved and non-evolved viruses, and between evolved viruses under different transmission modes for the three CMV strains (*F_3,135_*≥7.53;*P*≤1×10^−5^). In general, evolved viruses of the three strains showed lower virus accumulation than non-evolved viruses (*P*≤0.024), with the exception of horizontally evolved Fny-CMV (*P* = 0.378) (Table 2). In addition, the effect of the mode of transmission (vertical, horizontal or alternated) varied depending on the CMV strain. In Fny-CMV, virus accumulation was similar for vertically and alternately evolved viruses (*P* = 0.154), and in both cases was significantly lower than for horizontally evolved viruses (*P*≤0.001). In LS-CMV, the virus accumulation of vertically evolved viruses was lower than that of horizontally and alternately evolved viruses (*P*<1×10^−4^), which were not significantly different (*P* = 0.898) (Table 2). The mode of transmission did not affect the accumulation of De72-CMV (*P*≥0.200) (Table 2).

**Table 2:** Estimates of virus accumulation, virus effects on vegetative and reproductive growth, virulence, and seed transmission rate in the ‘original’ stock Cen-1 plants for three CMV strains passaged five times by strict vertical, strict horizontal and alternating vertical and horizontal transmission, plus non-evolved strains.

Strain	Transmission mode	Virus Accumulation^1^	Vegetative growth^2^: *RW_i_/RW_m_*	Reproductive growth^2^: *IW_i_/IW_m_*	Virulence^3^: *1−(SW_i_/SW_m_)*	% Seed Transmission^4^
Fny-CMV	Non-evolved	4.81±0.34	0.40±0.19	0.33±0.05	0.75±0.05	3.12±0.44
	Vertical	**3.85±0.22^(L)^**	0.59±0.11	**0.48±0.04^(H)^**	**0.58±0.06^(L)^**	**12.30±2.80^(H)^**
	Horizontal	5.19±0.37	0.45±0.05	0.34±0.02	0.74±0.03	-
	Alternate	**3.17±0.13**	0.56±0.13	**0.46±0.05^(H)^**	**0.56±0.04^(L)^**	-
LS-CMV	Non-evolved	14.16±1.13	0.24±0.06	0.27±0.10	0.76±0.04	1.76±0.40
	Vertical	**8.54±0.04^(L)^**	0.41±0.06	**0.50±0.04^(H)^**	**0.60±0.03^(L)^**	**11.47±2.49^(H)^**
	Horizontal	**11.74±0.21^(L)^**	0.33±0.04	0.29±0.03	0.78±0.03	-
	Alternate	**11.84±0.19^(L)^**	0.36±0.07	**0.40±0.03^(H)^**	**0.62±0.04^(L)^**	-
De72-CMV	Non-evolved	1.43±0.25	0.50±0.18	0.42±0.05	0.68±0.06	6.93±1.08
	Vertical	**1.07±0.03^(L)^**	0.52±0.06	**0.55±0.05^(H)^**	0.60±0.04	5.67±0.38
	Horizontal	**0.78±0.00^(L)^**	0.46±0.05	0.38±0.02	0.70±0.03	-
	Alternate	**0.81±0.03^(L)^**	0.49±0.05	0.42±0.05	0.61±0.05	-

1 Accumulation of virus RNA (µg/g fresh weight) estimated for 1∶1 mix of inoculated and systemically infected leaves.

2 Effect of CMV infection on rosette weight (*RW*) and inflorescence weight (*IW*) estimated as Trait*_i_*/Trait*_m_*, where *i* and *m* denote infected and mock-inoculated plants, respectively.

3 Virulence estimated as one minus the ratio of seed weight in infected *vs.* mock-inoculated plants: 1−(*SW*_i_ */SW*_m_).

4 Seed transmission rate estimated as the number of infected seedlings out of 100 seeds.

Values are mean±standard error of the 5 lineages in evolved viruses and 10 replicates in non-evolved strains.

Bold numbers indicate significant differences between non-evolved and evolved viruses. (L): Value in evolved viruses is lower than that in non-evolved viruses; (H) Value in evolved viruses is higher than that in non-evolved viruses.

The mode of transmission did not significantly affect the effect of infection on vegetative growth (*RW* ratio) of any CMV strain (Table 2) (*F_3,135_*≤0.73; *P*≥0.538). The effect of virus infection on reproductive growth (*IW* ratio) (Table 2) varied significantly among transmission modes (*F_3,135_*≥3.29; *P*≤0.023). In Fny-CMV and LS-CMV, the *IW* ratio was significantly higher in vertically and alternately evolved viruses than in horizontally evolved and non-evolved viruses (*P*≤0.051) (Table 1). In De72-CMV, the effect of infection on the *IW* ratio was significantly lower in vertically evolved viruses than in the other three treatments (*P*≤0.017), which were not significantly different (*P*>0.645) (Table 2). The effect of infection by Fny-CMV and LS-CMV on seed weight (i.e., virulence) significantly differed among transmission modes (*F_3,135_*≥5.15; *P*≤0.002). In both strains, virulence was lower when viruses were vertically and alternately evolved than when they were horizontally evolved or non-evolved (*P*≤0.030). No differences in virulence were observed between evolved and non-evolved De72-CMV viruses regardless of transmission mode (*F_3,135_* = 1.36; *P* = 0.260).

Finally, Fny-CMV and LS-CMV viruses had significantly higher vertical transmission rates when vertically evolved than non-evolved viruses in plants from the ‘original’ stock (*F_1,42_*≥4.42; *P*≤0.043), while no differences were observed for De72-CMV (*F_1,33_* = 1.15; *P* = 0.289) (Table 2).

Thus, CMV evolution under strict vertical transmission results in decreased virus accumulation and virulence. Vertical transmission also results in a reduced effect of infection on plant growth, particularly of the plant reproductive structures. These effects of passaging are higher in Fny-CMV and LS-CMV than in De72-CMV.

### Comparison of virus accumulation and virulence of evolved and non-evolved CMV strains in plants derived from the fifth vertical passage

Serial passages under strict vertical transmission may result not only in virus evolution, but also in host adaptation; that is, we might be selecting for plant individuals that transmit the virus to seed at higher rates. To analyse this possibility, seeds from non-infected plants of the fifth vertical passage representing plant lineages evolved with each of the three virus strains were grown and inoculated with the corresponding evolved lineages and with the non-evolved CMV isolates. In these plants, virus accumulation and effects of infection on plant growth (*RW* and *IW*) and on fecundity (*SW*) were analysed as in ‘original’ stock plants, but percentage of CMV seed transmission was not determined. For each combination of CMV strain and mode of transmission, the different virus lineages did not differ in any of the parameters estimated (*F*≤2.45; *P*≥0.060) (Table S2), and therefore lineage was not considered as a factor. As above, GLM analyses using ‘lineage’ as a nested factor did not alter the results.

Virus accumulation in evolved plants differed among treatments (*F_3,200_*≥3.59; *P*≤0.015). Fny-CMV and LS-CMV evolved viruses accumulated at lower levels than non-evolved viruses (*P*<1×10^−4^), while the opposite was observed in De72-CMV (*P*≤0.055) (Table 3). The effect of the mode of transmission varied depending on the CMV strain. In Fny-CMV, virus accumulation of vertically evolved viruses was lower than that of alternately evolved viruses (*P* = 0.001), with intermediate accumulation values of horizontally evolved viruses (*P*≤0.005) (Table 3). In LS-CMV, virus accumulation was not significantly different in vertically and alternately evolved viruses (*P* = 0.446), but was significantly less in horizontally evolved viruses (*P*<1×10^−4^). The mode of transmission did not affect the accumulation of De72-CMV (*P*≥0.216) (Table 3).

**Table 3:** Estimates of virus accumulation, virus effects on vegetative and reproductive growth, and virulence in horizontally inoculated Cen-1 plants derived from the fifth vertical transmission passage.

Strain	Transmission mode	Virus Accumulation^1^	Vegetative growth^2^: *RW_i_/RW_m_*	Reproductive growth^2^: *IW_i_/IW_m_*	Virulence^3^: *1−(SW_i_/SW_m_)*
Fny-CMV	Non-evolved	↑10.21±0.79	↓0.28±0.03	↓0.25±0.02	↑0.81±0.02
	Vertical	↓2.56±0.12	↑0.89±0.07	↑0.50±0.02	↓0.53±0.04
	Horizontal	5.38±0.09	0.50±0.02	0.33±0.01	↑0.81±0.02
	Alternate	7.13±0.36	0.60±0.03	0.39±0.02	↓0.75±0.02
LS-CMV	Non-evolved	↑19.82±0.86	0.24±0.02	0.32±0.02	↑0.79±0.01
	Vertical	↓8.28±0.18	↑0.50±0.04	↑0.55±0.02	↓0.55±0.02
	Horizontal	11.82±0.16	0.29±0.02	↑0.38±0.02	0.75±0.01
	Alternate	8.78±0.21	0.39±0.03	0.41±0.02	↑0.70±0.02
De72-CMV	Non-evolved	↓1.00±0.04	0.48±0.02	0.45±0.02	↑0.71±0.02
	Vertical	1.10±0.04	↑0.69±0.02	↑0.61±0.02	↓0.43±0.02
	Horizontal	↑1.14±0.02	0.46±0.04	0.41±0.02	0.73±0.01
	Alternate	↑1.08±0.02	0.57±0.06	0.43±0.02	0.68±0.01

1 Accumulation of virus RNA (µg/g fresh weight) estimated for 1∶1 mix of inoculated and systemically infected leaves.

2 Effect of CMV infection on rosette weight (*RW*) and inflorescence weight (*IW*) estimated as Trait*_i_*/Trait*_m_*, where *i* and *m* denote infected and mock-inoculated plants, respectively.

3 Virulence estimated as one minus the ratio of seed weight in infected *vs.* mock-inoculated plants: 1−(*SW*_i_ */SW*_m_).

Values are mean±standard error of the 5 lineages in evolved viruses and 10 replicates in non-evolved strains.

Large arrows indicate values significantly higher (↑) or lower (↓) than the equivalent value in Table 2 (*P*≤0.05), and small arrows indicate non-significant trends (*P*<0.10).

Differences in the effect of CMV infection in *RW* and *IW* were observed among the different modes of transmission (*F_3,200_*≥6.27; *P*<1×10^−4^), and followed similar patterns in the three virus strains (Table 3). In Fny-CMV and LS-CMV, vertically evolved viruses had significantly higher *RW* and *IW* ratios than the non-evolved viruses (*P*<1×10^−4^), with intermediate values for horizontally and alternately evolved viruses (Table 3). In De72-CMV the effect of infection on *RW* and *IW* ratios was less in vertically evolved viruses than in the other three treatments (*P*≤0.041), which were not significantly different among themselves (*P*≥0.068) (Table 2). In all strains, virulence significantly differed among modes of transmission (*F_3,200_*≥21.17; *P*<1×10^−4^), with vertically evolved viruses always being less virulent than viruses in the other three treatments (*P*<1×10^−4^). In addition, virulence was significantly lower in alternately evolved viruses than in the other two treatments (*P*≤0.052) (Table 3).

Finally, we explored if there were differences in virus accumulation and virulence of vertically evolved viruses in plants derived from the fifth vertical passage with respect to whether they were infected by vertical transmission (via seed) or horizontal transmission (mechanical inoculation). To do so, we compared the data above for plants mechanically inoculated (Table 3) with results for these traits in vertically infected plants derived from the fifth vertical passage (Table 1). Virus accumulation was higher in the horizontally inoculated than in the vertically infected plants for lineages of the three strains combined (*F_1,333_*≥450.11; *P*<1×10^−5^), and for each strain individually (*F_1_*≥720.45; *P*<1×10^−5^). Accordingly, virulence was always lower in vertically infected plants (*F_1_*≥12.58; *P*≤0.001).

In summary, CMV evolution under vertical transmission decreases virus accumulation, virulence and effect of infection on the growth of plants derived from the fifth vertical passage. Differences among modes of transmission were larger in these plants than in those from the ‘original’ stock for most traits, which suggests that plants changed during the vertical passages by increasing their resistance to virus infection. Whether these changes co-evolved with those observed in the viruses is analysed in the next section.

### Comparison of virus accumulation and virulence in ‘original’ stock plants and in plants derived from the fifth vertical passage

If vertical transmission of CMV resulted in selection for *Arabidopsis* plants with better performance under virus infection, the effect of infection with evolved and non-evolved strains would differ between ‘original’ stock plants and plants derived from the fifth vertical passage. To test this hypothesis, virus accumulation, *RW* and *IW* ratios and virulence in ‘original’ stock plants were compared with plants derived from the fifth vertical passage (Tables 2–3). Vertical transmission rate was compared between plants of the ‘original’ seed stock (Table 2) and plants of the fifth vertical passage (fifth passage in Figure 2).

Fny-CMV and LS-CMV vertically evolved viruses accumulated to lower levels (*F_1,100_*≥3.97;*P*≤0.054), and infected plants had higher *RW* and *IW* ratios, and lower virulence (*F_1,100_*≥3.61;*P*≤0.068) in plants derived from the fifth vertical passage than in ‘original’ stock plants (Table 3). The opposite was observed in the non-evolved viruses of these two strains (*F_1,100_*≥7.23; *P*≤0.009, for virus accumulation; and *F_1,100_*≥1.65; *P*≤0.085, for *RW* and *IW* ratios, and virulence). The exception was that no differences in *RW* and *IW* ratios were observed between LS-CMV-infected plants of the ‘original’ stock and those derived from the fifth vertical passage (*F_1,100_*≤1.68; *P*≥0.198) (Table 3). For De72-CMV, an increase of *RW* and *IW* ratios, and a reduction in virulence was found in plants derived from the fifth vertical passage relative to ‘original’ stock plants infected with vertically evolved viruses (*F_1,80_*≥5.36; *P*≤0.023), but virus accumulation was similar in both types of plants (*F_1,80_* = 0.34; *P* = 0.560). Non-evolved viruses were marginally more virulent in plants derived from the fifth vertical passage than in ‘original’ stock plants (*F_1,80_*≥2.97; *P*≤0.090) (Table 3). Vertical transmission was higher in plants of the fifth passage of vertical transmission than in plants of the ‘original’ stock (compare Figure 2 and Table 2; *F*≥6.93; *P*≤0.013).

Fny-CMV and LS-CMV strains horizontally evolved (Table 3) caused less reduction in *IW* and were more virulent in plants derived from the fifth vertical passage compared with ‘original’ stock plants (*F_1,100_*≥4.69; *P*≤0.033). In De72-CMV virus accumulation was higher in plants derived from the fifth vertical passage than in the ‘original’ stock plants (*F_1,80_* = 301.33; *P*≤1×10^−4^).

Finally, Fny-CMV and LS-CMV alternately evolved were more virulent in plants derived from the fifth vertical passage compared with ‘original’ stock plants (*F_1,100_*≥4.40; *P*≤0.040), but virus accumulation of De72-CMV was higher in ‘original’ stock plants than in plants derived from the fifth vertical passage (*F_1,80_* = 41.92; *P*≤1×10^−4^). No significant differences were found in the rest of comparisons (Tables 1–3).

Thus, differences in the traits analysed between ‘original’ stock plants and plants derived from the fifth vertical passage indicate that the latter group of plants have mechanisms to reduce virus accumulation, the effects of infection on plant growth and on fecundity (virulence) by vertically transmitted viruses, often at the cost of increased virus accumulation and/or virulence of non-evolved viruses. These results support the hypothesis of plant-virus co-evolution during vertical transmission passages.

## Discussion

Most experimental analyses of virulence evolution are based on the hypothesis that virulence, which is correlated with parasite multiplication, is determined by trade-offs with parasite transmission rate [2], [10]–[12]. However, the factors that modulate this trade-off and how they act are only partially understood. Although theory has identified several of these potential modulators, their effect has seldom been analysed experimentally. Here we investigated the role of two such major factors in the evolution of virulence: the mode of transmission, and host adaptation in response to parasite evolution [8], [14], [16], [22]. The paucity of information on this subject has been attributed, in part, to the lack of suitable experimental systems [2]. For our experiments, we used the plant virus CMV and its host plant *Arabidopsis thaliana*, a system with several traits suitable for our objectives: i) CMV is a pathogen of *Arabidopsis* that is found at high incidence in *Arabidopsis* wild populations [45], ii) CMV is transmitted both horizontally and vertically in *Arabidopsis* [45; unpublished data], iii) *Arabidopsis* has a short generation time, which allows serial passages of vertical transmission to be performed in a reasonable time frame; and iv) recovery of *Arabidopsis* plants to CMV infection has not been described. From the perspective of viral fitness, host recovery is equivalent to host death in which the virus can no longer replicate or be transmitted. Therefore, recovery would affect the transmission rate, potentially blurring the virulence-transmission trade-off [54]. We serially passaged three CMV strains in *Arabidopsis* under strict vertical or strict horizontal transmission, and quantified virulence and traits related to viral fitness in the evolved and non-evolved viruses. We used the effect of virus infection on plant fecundity as a measure of virulence. Virulence encompasses the negative effect of a parasite on host longevity and fecundity [8], [55], [56]. In vertically transmitted parasites, the effect of infection on host fecundity is the most relevant trait for transmission success [10], [13], [14], [23]–[25]. Thus, host fecundity is the best proxy for virulence to analyse the vertical transmission-virulence trade-off. However, longevity of hosts infected with horizontally transmitted parasites, which is linked to the length of the infectious period, is the most obvious determinant of parasite transmission rate. Indeed, in such parasites longevity of infected hosts has been proposed to be a good proxy for virulence [57]. Although CMV infection significantly affects host fecundity, it has little effect on the lifespan of *Arabidopsis* [41]. This mimics the effect of sterilizing parasites on their hosts. For sterilizing parasites horizontal transmission may be correlated with host fecundity, in which case the effect on host fecundity is the best proxy for virulence [8], [55], [58].

As predicted by theory, our results show a key role of the mode of transmission in the evolution of virulence. The comparison of non-evolved viruses with those evolved under strict vertical transmission indicated that the latter increased their rate of vertical transmission, and that adaptation to this transmission mode was associated with the reduction of virus multiplication and virulence. In contrast, evolution under strict horizontal transmission did not result in changes of virus multiplication or virulence. We did not quantify the rate of vertical transmission of horizontally evolved lineages. However, the absence of evolution in virus multiplication and in virulence of horizontally passaged viruses − two traits that are correlated with vertical transmission rate in our system − may be suggestive of a lack of change in vertical transmission rate. Thus, our results support predictions of the models of virulence evolution based on the trade-off hypothesis [14], [16], [22] in that we found a negative correlation between rate of vertical transmission and virulence. This negative correlation was also found in previous studies with bacteria and insect parasites [26]–[28], [30]–[33], [59], [60], and in the only other study of a plant virus, *Barley stripe mosaic virus* (BSMV) in barley [29]. Predictions of the trade-off hypothesis were not supported by results of the only other reported analyses of a plant-parasite system, in which the rate of vertical transmission was positively correlated with virulence. However, this is an unusual system as it involves a sterilizing fungus. This fungus invades the plant reproductive structures, and more virulent fungal strains have better access to the seeds. Thus, more virulent strains compensate the higher reduction of plant fecundity by infecting more seeds than less virulent strains [34], [35]. More plant-parasite systems need to be characterized to determine the generality of the trade-off predictions.

Perhaps one of the most striking results of this work is the observed negative correlation between virus multiplication and transmission rate. In other plant-parasite systems higher parasite load is associated with higher percentage of infected seeds [29], [34], [35]. Limited knowledge on the mechanisms of CMV seed transmission hinders the interpretation of this result. CMV is present in the embryo, the endosperm, and the coat of infected seeds [61]; and the virus is thought to gain access to seed tissues either through the ovules or pollen [62], or through the suspensor that connects the mother plant and the developing seed [63]. Hence, vertical transmission rate would be determined by the capacity of the virus to reach the seed during gametogenesis and/or while the suspensor is still functional, and by the ability of plant defense to block virus access to the seed. If this model holds for CMV and *Arabidopsis*, a negative correlation between virus multiplication and transmission rate would be explained: 1) if lower virus titer would result in a less efficient triggering of plant defenses that prevent seed infection; and/or 2) if serial passages of vertical transmission selects for virus variants with mutations that facilitate direct or indirect access for CMV to the seed even at low multiplication levels.

Virulence and within-host multiplication were positively correlated in viruses evolved under strict vertical transmission in this study, consistent with the central assumption of the trade-off hypothesis [2], [10]–[12]. This result is at odds with observations in BSMV and barley where neither vertical transmission nor virulence correlated with virus multiplication [29], [64]. Virulence and within-host multiplication does not correlate across CMV and *Arabidopsis* genotypes, however, as genotype-specific host tolerance to virus infection uncoupled both traits [65]. Interestingly, the *Arabidopsis* genotype used in the present work (Cen-1) was rated as a low-tolerance genotype [41], so that the relationship between virulence and within-host multiplication will not be blurred by tolerance. Note also that the relationship between virulence and within-host multiplication is not linear (Figures 2–3). Thus, it is possible that this relationship could go unnoticed in some studies [66] if data are within the range in which this relationship is saturated.

The reduction of CMV virulence associated with increased rates of vertical transmission is also associated with smaller effects of infection in both the vegetative and reproductive efforts of the host (*RW* and *IW*). Vertically transmitted parasites may select for modifications of host life-history traits that enhance host reproductive success and parasite transmission. For instance, insects have increased survival when infected by bacteria such as *Rickettsia* or *Wolbachia* [67], [68], or increased reproductive time span when infected by microsporidian parasites [69]. The higher *RW* and *IW* ratios of *Arabidopsis* infected with vertically evolved viruses compared with non-evolved viruses might be interpreted similarly, as *Arabidopsis* fecundity is positively correlated with both the vegetative and the reproductive growth of the plant [41]. Thus, the observed evolution towards lower virulence may represent a selective advantage for the virus, as it increases the chances for its vertical transmission.

In certain contexts, the parasite and its host may have sufficient alignment of interests to boost host-virus co-evolution: Parasite infection may be still detrimental for the host, but maximization of host fitness upon infection also maximizes the parasite fitness [70]. Such maximization might occur when parasites adapt to vertical transmission [3], [71], [72]. In this scenario, the host may also evolve to increase the parasite's rate of vertical transmission [39], [73], a possibility that has seldom been analysed [36]. Our data provide evidence that this may be the case for the *Arabidopsis*-CMV interaction, as vertical transmission rate was higher, and virulence was lower, in plants selected during serial passages of vertical transmission as compared with plants from the ‘original’ stock. Although these changes in the passaged plant were not enough to completely compensate the negative effect of CMV infection in plant fitness, they significantly increased plant fecundity as compared with ‘original’ stock plants. The observed changes in the host plant during vertical passages are related to increased resistance, i.e., reduced parasite multiplication [74]. Resistance was particularly effective when infection was the result of vertical transmission compared with horizontal transmission (Tables 1–3), although we cannot rule out that this would reflect that plants infected horizontally may be weaker as they come from seeds already challenged (unsuccessfully) with CMV. In either case, a degree of plant resistance may paradoxically represent a benefit for CMV strains adapted to vertical transmission, as long as multiplication does not go below a threshold that importantly decreases seed transmission rate. These patterns of plant-virus co-evolution may be due to selection during passages. In wild *Arabidopsis* populations in Spain, the inbreeding coefficient, i.e., probability of autozygosity [75], ranges between 0.87 and 0.99 [76]. Hence, wild accessions are not homozygous at all loci, and genetic variation may be expected in an experimental plant population derived from a single individual. Therefore, selection may have acted upon genetic variation in the original stock plant. Evolution in the *Arabidopsis*-CMV interaction is compatible with conditions required for host-parasite co-evolution as defined by [37] in which hosts and pathogens exert reciprocal selection on each other. Additionally, we can speculate on genetic changes induced by virus infection, resulting either in genomic rearrangements or in epigenetic variation [77], [78] that may explain the observed changes in the host plant. Since all passages of horizontal transmission were done in plants from the ‘original’ stock, we cannot address whether plant adaptation to horizontally passaged viruses occurred in our experiments.

The hypothesis that adaptation to vertical transmission is advantageous for both the virus and the plant is in apparent contradiction with the observation that vertical transmission of CMV occurs only at low rates across *Arabidopsis* genotypes [45; this study]. However, adaptation in *Arabidopsis* came at the cost of increased virulence and increased multiplication of the non-evolved viruses, i.e., a trade-off in host fitness when infected with non-evolved virus genotypes. In natural populations of *Arabidopsis*, CMV spreads both by horizontal and vertical transmission [45; unpublished data], and it is reasonable to hypothesize that both the optimal level of vertical transmission for the host plant, and of virulence for the virus would be influenced by the observed adaptation trade-off. In nature, most virus genotypes are likely to be better adapted to horizontal than to vertical transmission, and plant genotypes adapted to vertical transmission would suffer from extra fitness penalties when infected by most virus genotypes.

Thus, virulence evolution could not be explained only by trade-offs between the relative rates of vertical and horizontal transmission, as stated by the ‘continuum hypothesis’: our results illustrate the key role that the complex interplay between mode of transmission and host-parasite co-evolution has in determining virulence evolution. This type of interplay should be considered in theoretical and experimental analyses of virulence evolution, and in the design of better strategies for virulence management.

## Materials and Methods

### Virus isolates and *Arabidopsis* genotype

Three virus strains were used: Fny-CMV and De72-CMV belonging to subgroup I of CMV isolates, and LS-CMV, belonging to subgroup II. Fny-CMV and LS-CMV are well characterized and were derived from biologically active cDNA clones [79], [80] by *in vitro* transcription with T7 RNA polymerase (New England Biolabs, Ipswich, MA, USA). De72-CMV was obtained from a field-infected plant of *Diplotaxis erucoides* (Brassicaceae), a host closely related to *Arabidopsis* [81]. Transcripts of Fny-CMV and LS-CMV, and purified viral RNA from De72-CMV were used to infect tobacco (*Nicotiana tabacum*) plants for virus multiplication. CMV virions from tobacco leaves were purified as described in [82], and viral RNA was extracted by virion disruption with phenol and sodium dodecyl sulphate.

Accession Cen-1 (Centenera, Spain) was selected from a panel of eighteen *Arabidopsis* accessions as it combined a higher efficiency of CMV vertical transmission − ranging between 2–8% depending on the virus isolate [unpublished data] −, with a relatively short life cycle [65]. For plant growth, seeds of Cen-1 were sown on filter paper soaked with water in single plastic Petri dishes, and stratified in darkness at 4°C for five days before transferring for germination to a growth chamber (22°C, 14 h light and 70% relative humidity). Five day-old seedlings were planted in soil in 10.5-cm-diameter pots (0.43 l volume), and grown in a greenhouse (25/20°C day/night, 16 h light).

### Serial passages

The three CMV isolates were serially passaged in Cen-1 plants five times by strict vertical transmission, strict horizontal transmission, or alternating both modes of transmission (Figure 1).

To analyse virus evolution under strict vertical transmission, Cen-1 plants from a seed stock (referred to as ‘original’ stock) generously provided by Dr. Carlos Alonso-Blanco (CNB-CSIC, Spain) were mechanically inoculated with purified CMV RNA of the three CMV strains (100 ng/ml) in 0.1 M Na_2_HPO_4_ when rosettes had 4–5 leaves (stages 1.04–1.05 in [50]) with five replicates per treatment. Seeds from each infected plant were harvested at complete senescence (stage 9.0 as in [50]) generating five independent vertically transmitted CMV lineages per virus strain. One hundred Cen-1 seeds per CMV-infected plant were grown as described above, and CMV infection in twenty-day-old plants was detected by dot-blot hybridization (see below) to estimate the percentage of seed transmission. Infected plants were allowed to complete their life cycle and seeds were harvested at senescence. One infected plant per CMV lineage was randomly selected to start the next plant generation, and the process was repeated for five generations; i.e., five passages of vertical transmission (Figure 1). The percentage of seed transmission was determined in every passage as described above. Seeds from uninfected individuals of the fifth generation were also harvested.

To analyse virus evolution under strict horizontal transmission, sap extracts from the fifteen Cen-1 plants inoculated to generate the vertically transmitted lineages were used to mechanically inoculate fifteen uninfected Cen-1 plants from the ‘original’ Cen-1 seed stock; these fifteen plants represented the first passage of horizontal transmission in five independent lineages for each virus strain. Sap extract from each of these fifteen infected plants was used to inoculate ten plants at stages 1.04–1.05 [50] again from the ‘original’ Cen-1 seed stock. CMV infection was detected fifteen days post-inoculation (dpi) by dot-blot hybridization (see below). Sap extract from one randomly chosen infected plant per lineage was used to inoculate ten new plants of the ‘original’ stock of Cen-1 seeds. This procedure was repeated for five passages (Figure 1).

In parallel, five viral lineages per CMV strain were evolved alternating vertical and horizontal transmission to generate five alternately transmitted lineages. To do so, 100 seeds from each of the fifteen plants used to generate the vertically transmitted CMV lineages were grown, and CMV infection was detected, as described above. Sap extracts from one infected plant per lineage were used to inoculate ten Cen-1 plants from the ‘original’ seed stock. The procedure for this treatment to this point is identical to that used for the strict horizontal treatment. However, seeds from these horizontally infected plants were harvested at complete senescence. One hundred seeds per plant were grown, virus infection was detected, and one plant per lineage was randomly chosen to generate the new vertically transmitted generation. This process was repeated until the third horizontal passage was completed, for a total of three vertical passages and three horizontal passages (Figure 1).

### Analysis of the evolution of virulence in evolved CMV lineages

Multiplication and virulence of non-evolved and evolved CMV strains after vertical, horizontal and alternated passages, referred to as vertically evolved, horizontally evolved and alternately evolved viruses, were analysed in plants from the ‘original’ seed stock and in plants derived from the fifth vertical transmission passage. Sap extracts from CMV-infected plants of the fifth vertical and horizontal passages were used to inoculate ten Cen-1 plants from the ‘original’ seed stock and ten plants from seeds from the fifth passage of vertical transmission per virus lineage. Only five plants were inoculated for each of the alternately evolved viruses. In the fourth vertical transmission passage, no infected seeds were detected for one of the De72-CMV lineages; therefore, only four replicates were analysed for this treatment. Plants derived from the fifth passage of vertical transmission were chosen randomly to represent plants passaged with each of the three CMV strains, and inoculated with evolved lineages of the corresponding strain. In addition, ten plants of the ‘original’ Cen-1 stock and ten plants from one randomly chosen uninfected replicate after five vertical passages per each of the three strains were inoculated with sap from Cen-1 plants infected with non-evolved Fny-CMV, De72-CMV and LS-CMV. Mock-inoculated plants, with ten replicates per seed stock, were included as a control. Virus multiplication, and effect of virus infection on rosette and inflorescence growth, and on seed production were determined in each plant as described below.

### Quantification of CMV multiplication

CMV multiplication was quantified as virus RNA accumulation. Total nucleic acid extracts from four leaf discs (0.01 g fresh weight) collected from four different rosette and inflorescence leaves were obtained using TRI-reagent (Sigma-Aldrich, St. Louis, MO, USA). RNA quantification was done by dot-blot hybridization with ^32^P-labeled RNA probes obtained by transcription from cDNA clones representing the 3′ non-coding region of the three genomic RNAs, which is highly similar within a CMV isolate. For Fny-CMV and De72-CMV, a probe representing nucleotides 1933 to 2215 of Fny-CMV RNA3 (GeneBank Acc. No. D10538) was used, and for LS-CMV the probe represented nucleotides 1861 to 2193 of LS-CMV RNA3 (Acc. No AF127976). Internal CMV standards for subgroup I (Fny-CMV or De72-CMV), and subgroup II (LS-CMV) were included as a two-fold dilution series of purified RNA (0.5 to 0.001 µg) in nucleic acid extracts from mock-inoculated *Arabidopsis* plants. RNA extracts from infected plants were blotted at different dilutions to ensure that hybridization signal was on the linear portion of the RNA concentration-hybridization signal curve. All hybridizations were done at 65°C overnight in 6× SSC, 5× Denhardt's mixture, 0.1% sodium dodecyl sulphate, and yeast tRNA at 50 mg/ml [83]. RNA hybridization signal was detected using a Typhoon 9400 scanner (GE Healthcare, Chalfont St. Giles, UK) after exposure of the Eu^+2^ store phosphor screens to the labelled samples, and CMV multiplication was quantified by using Image-Quant 5.2 (Molecular Dynamics, GE Healthcare) [84].

### Estimation of virulence and effect of CMV infection on plant growth

Virulence is defined as the negative effect of infection on host fitness [1], [2], a good proxy for host fitness being total fecundity. Our previous work showed that CMV infection does not affect the viability or weight of individual seeds in Cen-1 [56]; therefore we used total seed weight (*SW*) as a measure of host fitness. Thus, virulence was estimated as one minus the ratio of the total seed weight of infected (*SW_i_*) to total seed weight of mock-inoculated (*SW_m_*) plants, 1−(*SW_i_/SW_m_*). We also measured plant dry weight at complete senescence after drying at 65°C until constant weight, as a measure of tolerance (see Introduction). Rosette weight (*RW*) and inflorescence weight including seeds (*IW*) were measured separately. To quantify the effect of CMV infection on *RW* and *IW*, the value of each infected plant was divided by the mean value of the mock-inoculated plants (*Trait_i_*/*Trait_m_*, *i* and *m* denote infected and mock-inoculated plants, respectively).

### Estimation of seed transmission rate

In each vertical passage, the rate of seed transmission was determined as the percentage of infected individuals out of the 100 germinated plants per lineage. Two leaves of twenty-day-old plants were harvested and pooled in groups of ten individuals, and total nucleic acid extracts of these pools were obtained. We pooled leaves among plants because of the low percentage of plants infected [unpublished data]. The presence of CMV in each pool was detected by dot-blot hybridization with the same probes used for quantification of virus accumulation. As negative controls, total nucleic acid extracts from pools of ten twenty-day-old non-infected plants were used. Samples with hybridization signal more than two-fold higher the negative controls were considered as positive. Two leaves were harvested from each plant within each positive pool for detection of CMV individually by dot-blot hybridization as described above.

In the ‘original’ stock plants infected with the vertically evolved CMV strains, five infected plants per lineage were randomly chosen and 100 seeds per plant were grown as described above. Five-day-old seedlings were harvested in pools of ten individuals and the presence of CMV in each pool was detected essentially as described above. Because we pooled seedlings for this analysis, seed transmission rate was estimated assuming that the number of seedlings infected follows a Poisson distribution [85]. Estimates obtained assuming a binomial distribution yielded the same results.

### Statistical analyses

Data on virus multiplication, seed transmission rate, and rosette, inflorescence and seed weights, and their various transformations, including virulence, were homoscedastic and were analysed using full factorial General Linear Models (GLM). Virus strain/lineage, type of plant (stock *vs.* passaged), and mode of transmission were considered as fixed effect factors. Significance of differences among classes within each factor was determined by Least Significant Difference (LSD) analyses. Linear associations between virus multiplication, seed transmission rate and virulence during serial passages was analysed by bivariate tests and using Pearson's correlation test [85]. To investigate whether non-linear models better explained these associations, we fitted them to logarithmic, exponential and quadratic models [86]. Regression lines were compared using ANOVA to test the equality of slopes and intercepts; non-linear curves were log transformed for this analysis. All statistical analyses were performed using the statistical software packages SPSS 21.0 (SPSS Inc., Chicago, IL, USA).

## Supporting Information

Table S1 Estimates of virus accumulation, effect of infection in vegetative and reproductive growth, and virulence of each CMV lineage in ‘original’ Cen-1 plants.
(DOCX)

Table S2 Estimates of virus accumulation, effect of infection in vegetative and reproductive growth, and virulence for each lineage in Cen-1 plants derived from the fifth vertical transmission passage.
(DOCX)
