# Supplementary material for: Vertical Transmission Selects for Reduced Virulence in a Plant Virus and for Increased Resistance in the Host
Source: PLoS Pathog. 2014 Jul 31;10(7):e1004293. doi: 10.1371/journal.ppat.1004293 (PMC4117603; doi:10.1371/journal.ppat.1004293)
Supplement: Table S1 — Estimates of virus accumulation, effect of infection in vegetative and reproductive growth, and virulence of each CMV lineage in ‘original’ Cen-1 plants. (DOCX) [file ppat.1004293.s001.docx]

**Table S1.** Estimates of virus accumulation, effect of infection in vegetative and reproductive growth, and virulence of each CMV lineage in ‘original’ Cen-1 plants.

| **Strain** | **Transmission**  **Mode** | **Lineage** | **Virus**  **Accumulation^1^** | **Vegetative growth^2^:**  ***RW_i_/RW_m_*** | **Reproductive growth^2^:**  ***IW_i_/IW_m_*** | **Virulence^3^:**  ***1-(SW_i_/SW_m_)*** | **% Seed Transmission^4^** |
| --- | --- | --- | --- | --- | --- | --- | --- |
|  |  |  |  |  |  |  |  |
| Fny-CMV | Vertical | Fny.1 | 3.80±0.02 | 0.56±0.20 | 0.56±0.12 | 0.50±0.16 | 14.23±4.14 |
|  |  | Fny.2 | 3.13±0.00 | 0.53±0.14 | 0.41±0.09 | 0.54±0.06 | 10.04±1.94 |
|  |  | Fny.3 | 3.95±0.03 | 0.48±0.09 | 0.49±0.08 | 0.55±0.11 | 5.84±1.17 |
|  |  | Fny.4 | 3.93±0.00 | 0.56±0.09 | 0.45±0.08 | 0.61±0.10 | 19.13±1.25 |
|  |  | Fny.5 | 4.44±0.03 | 0.69±0.60 | 0.50±0.09 | 0.47±0.18 | 12.30±2.80 |
|  |  |  |  |  |  |  |  |
|  | Horizontal | Fny.1 | 5.42±0.16 | 0.40±0.09 | 0.33±0.05 | 0.64±0.09 | - |
|  |  | Fny.2 | 5.12±0.00 | 0.46±0.17 | 0.40±0.06 | 0.72±0.06 | - |
|  |  | Fny.3 | 5.43±0.23 | 0.39±0.06 | 0.32±0.04 | 0.79±0.07 | - |
|  |  | Fny.4 | 4.14±0.01 | 0.33±0.10 | 0.27±0.05 | 0.77±0.04 | - |
|  |  | Fny.5 | 6.05±0.05 | 0.49±0.13 | 0.40±0.04 | 0.78±0.04 | - |
|  |  |  |  |  |  |  |  |
|  | Alternate | Fny.1 | 3.77±0.06 | 0.57±0.10 | 0.46±0.05 | 0.65±0.09 | - |
|  |  | Fny.2 | 2.92±0.00 | 0.49±0.21 | 0.46±0.04 | 0.50±0.09 | - |
|  |  | Fny.3 | 2.93±0.03 | 0.55±0.06 | 0.47±0.05 | 0.54±0.12 | - |
|  |  | Fny.4 | 3.24±0.38 | 0.65±0.40 | 0.50±0.22 | 0.62±0.07 | - |
|  |  | Fny.5 | 2.92±0.01 | 0.64±0.21 | 0.40±0.10 | 0.53±0.10 | - |
|  |  |  |  |  |  |  |  |
|  | Non-evolved |  | 4.81±0.34 | 0.40±0.05 | 0.33±0.05 | 0.75±0.05 | 3.12±0.44 |
|  |  |  |  |  |  |  |  |
| De72-CMV | Vertical | De72.1 | 1.13±0.07 | 0.54±0.12 | 0.52±0.11 | 0.68±0.02 | 6.49±1.24 |
|  |  | De72.2 | 1.10±0.09 | 0.61±0.20 | 0.64±0.15 | 0.51±0.15 | 6.22±0.94 |
|  |  | De72.3 | 1.02±0.06 | 0.42±0.11 | 0.44±0.05 | 0.57±0.09 | 5.12±0.85 |
|  |  | De72.4 | 1.02±0.03 | 0.50±0.09 | 0.61±0.10 | 0.62±0.08 | 5.74±0.60 |
|  |  |  |  |  |  |  |  |
|  | Horizontal | De72.1 | 0.78±0.00 | 0.46±0.14 | 0.46±0.06 | 0.64±0.08 | - |
|  |  | De72.2 | 0.78±0.00 | 0.52±0.15 | 0.33±0.03 | 0.72±0.08 | - |
|  |  | De72.3 | 0.78±0.00 | 0.46±0.08 | 0.37±0.04 | 0.73±0.05 | - |
|  |  | De72.4 | 0.79±0.01 | 0.38±0.09 | 0.39±0.05 | 0.72±0.05 | - |
|  |  |  |  |  |  |  |  |
|  | Alternate | De72.1 | 0.77±0.00 | 0.49±0.09 | 0.41±0.15 | 0.58±0.13 | - |
|  |  | De72.2 | 0.88±0.13 | 0.54±0.12 | 0.42±0.06 | 0.58±0.10 | - |
|  |  | De72.3 | 0.83±0.04 | 0.45±0.10 | 0.39±0.05 | 0.68±0.08 | - |
|  |  | De72.4 | 0.78±0.00 | 0.50±0.12 | 0.44±0.13 | 0.60±0.08 | - |
|  |  |  |  |  |  |  |  |
|  | Non-evolved |  | 1.43±0.25 | 0.50±0.18 | 0.42±0.05 | 0.68±0.08 | 6.93±1.08 |
|  |  |  |  |  |  |  |  |
| LS-CMV | Vertical | LS.1 | 8.45±0.08 | 0.36±0.10 | 0.52±0.06 | 0.54±0.08 | 20.52±10.32 |
|  |  | LS.2 | 8.51±0.05 | 0.41±0.03 | 0.42±0.05 | 0.53±0.08 | 6.43±1.99 |
|  |  | LS.3 | 8.39±0.03 | 0.30±0.15 | 0.46±0.05 | 0.64±0.04 | 12.63±4.76 |
|  |  | LS.4 | 8.69±0.08 | 0.45±0.12 | 0.58±0.13 | 0.70±0.04 | 12.29±5.42 |
|  |  | LS.5 | 8.66±0.12 | 0.51±0.18 | 0.51±0.06 | 0.59±0.07 | 5.27±1.02 |
|  |  |  |  |  |  |  |  |
|  | Horizontal | LS.1 | 11.17±0.38 | 0.31±0.08 | 0.27±0.05 | 0.77±0.04 | - |
|  |  | LS.2 | 10.53±0.31 | 0.34±0.08 | 0.26±0.05 | 0.85±0.03 | - |
|  |  | LS.3 | 10.53±0.10 | 0.34±0.08 | 0.32±0.07 | 0.73±0.07 | - |
|  |  | LS.4 | 13.27±0.01 | 0.28±0.06 | 0.22±0.04 | 0.75±0.08 | - |
|  |  | LS.5 | 13.30±0.01 | 0.39±0.11 | 0.39±0.07 | 0.76±0.04 | - |
|  |  |  |  |  |  |  |  |
|  | Alternate | LS.1 | 11.45±0.54 | 0.45±0.21 | 0.44±0.09 | 0.66±0.13 | - |
|  |  | LS.2 | 12.44±0.35 | 0.34±0.07 | 0.32±0.08 | 0.72±0.09 | - |
|  |  | LS.3 | 11.99±0.45 | 0.32±0.17 | 0.48±0.06 | 0.51±0.10 | - |
|  |  | LS.4 | 12.28±0.36 | 0.26±0.04 | 0.42±0.04 | 0.57±0.10 | - |
|  |  | LS.5 | 11.04±0.20 | 0.37±0.14 | 0.33±0.03 | 0.73±0.04 | - |
|  |  |  |  |  |  |  |  |
|  | Non-evolved |  | 14.16±1.13 | 0.24±0.06 | 0.27±0.10 | 0.79±0.04 | 1.76±0.40 |

^1^ Accumulation of virus RNA (μg/g fresh weight) estimated for 1:1 mix of inoculated and systemically infected leaves.

^2^ Effect of CMV infection on rosette weight (*RW*) and inflorescence weight (*IW*) estimated as Trait*_i_*/Trait*_m_*, where *i* and *m* denote infected and mock-inoculated plants, respectively.

^3^ Virulence estimated as one minus the ratio of seed weight in infected *vs.* mock-inoculated plants: 1-(*SW*_i_*/SW*_m_).

^4^ Seed transmission rate estimated as the number of infected seedlings out of 100 seeds.

Values are mean±standard error of 10 replicates.
