# Supplementary material for: Vertical Transmission Selects for Reduced Virulence in a Plant Virus and for Increased Resistance in the Host
Source: PLoS Pathog. 2014 Jul 31;10(7):e1004293. doi: 10.1371/journal.ppat.1004293 (PMC4117603; doi:10.1371/journal.ppat.1004293)
Supplement: Table S2 — Estimates of virus accumulation, effect of infection in vegetative and reproductive growth, and virulence for each lineage in Cen-1 plants derived from the fifth vertical transmission passage. (DOCX) [file ppat.1004293.s002.docx]

**Table S2.** Estimates of virus accumulation, effect of infection in vegetative and reproductive growth, and virulence for each lineage in Cen-1 plants derived from the fifth vertical transmission passage.

| **Strain** | **Transmission**  **Mode** | **Lineage** | **Virus**  **Accumulation^1^** | **Vegetative growth^2^:**  ***RW_i_/RW_m_*** | **Reproductive growth^2^: *IW_i_/IW_m_*** | **Virulence^3^:**  **1-*(SW_i_/SW_m_)*** |
| --- | --- | --- | --- | --- | --- | --- |
|  |  |  |  |  |  |  |
| Fny-CMV | Vertical | Fny.1 | 2.13±0.00 | 0.86±0.13 | 0.51±0.06 | 0.59±0.13 |
|  |  | Fny.2 | 3.13±0.01 | 0.76±0.14 | 0.43±0.06 | 0.58±0.05 |
|  |  | Fny.3 | 3.24±0.10 | 0.78±0.12 | 0.51±0.04 | 0.52±0.07 |
|  |  | Fny.4 | 2.14±0.01 | 1.06±0.15 | 0.58±0.04 | 0.57±0.07 |
|  |  | Fny.5 | 2.17±0.02 | 1.00±0.17 | 0.46±0.03 | 0.67±0.04 |
|  |  |  |  |  |  |  |
|  | Horizontal | Fny.1 | 4.88±0.12 | 0.44±0.06 | 0.30±0.03 | 0.85±0.03 |
|  |  | Fny.2 | 5.11±0.01 | 0.55±0.04 | 0.40±0.04 | 0.80±0.04 |
|  |  | Fny.3 | 5.93±0.00 | 0.58±0.05 | 0.34±0.02 | 0.79±0.04 |
|  |  | Fny.4 | 5.86±0.24 | 0.35±0.04 | 0.29±0.03 | 0.83±0.02 |
|  |  | Fny.5 | 5.12±0.07 | 0.55±0.05 | 0.33±0.03 | 0.77±0.04 |
|  |  |  |  |  |  |  |
|  | Alternate | Fny.1 | 6.71±0.08 | 0.49±0.05 | 0.37±0.04 | 0.75±0.04 |
|  |  | Fny.2 | 6.37±0.09 | 0.68±0.06 | 0.42±0.04 | 0.74±0.05 |
|  |  | Fny.3 | 6.21±0.27 | 0.58±0.07 | 0.37±0.04 | 0.74±0.04 |
|  |  | Fny.4 | 6.80±0.04 | 0.64±0.12 | 0.42±0.05 | 0.72±0.04 |
|  |  | Fny.5 | 9.58±0.62 | 0.62±0.07 | 0.39±0.03 | 0.79±0.03 |
|  |  |  |  |  |  |  |
|  | Non-evolved | NOEV.1 | 10.56±2.37 | 0.26±0.06 | 0.31±0.09 | 0.83±0.04 |
|  |  | NOEV.2 | 11.75±2.38 | 0.27±0.07 | 0.24±0.05 | 0.84±0.03 |
|  |  | NOEV.3 | 9.68±1.38 | 0.27±0.04 | 0.26±0.03 | 0.79±0.03 |
|  |  | NOEV.4 | 10.08±0.40 | 0.33±0.11 | 0.25±0.03 | 0.77±0.07 |
|  |  | NOEV.5 | 8.98±1.60 | 0.28±0.03 | 0.20±0.02 | 0.83±0.01 |
|  |  |  |  |  |  |  |
| De72-CMV | Vertical | De72.1 | 1.19±0.10 | 0.79±0.03 | 0.64±0.04 | 0.41±0.08 |
|  |  | De72.2 | 1.05±0.07 | 0.65±0.04 | 0.58±0.04 | 0.45±0.05 |
|  |  | De72.3 | 1.22±0.04 | 0.70±0.05 | 0.60±0.02 | 0.46±0.04 |
|  |  | De72.4 | 0.95±0.05 | 0.63±0.05 | 0.61±0.05 | 0.39±0.04 |
|  |  |  |  |  |  |  |
|  | Horizontal | De72.1 | 1.09±0.04 | 0.47±0.07 | 0.47±0.05 | 0.70±0.03 |
|  |  | De72.2 | 1.09±0.05 | 0.51±0.05 | 0.39±0.04 | 0.72±0.02 |
|  |  | De72.3 | 1.18±0.03 | 0.43±0.14 | 0.38±0.03 | 0.74±0.02 |
|  |  | De72.4 | 1.18±0.03 | 0.44±0.06 | 0.42±0.06 | 0.75±0.04 |
|  |  |  |  |  |  |  |
|  | Alternate | De72.1 | 1.10±0.05 | 0.53±0.17 | 0.36±0.06 | 0.67±0.02 |
|  |  | De72.2 | 1.07±0.05 | 0.61±0.06 | 0.44±0.05 | 0.69±0.02 |
|  |  | De72.3 | 0.98±0.04 | 0.53±0.18 | 0.43±0.02 | 0.67±0.01 |
|  |  | De72.4 | 1.17±0.03 | 0.61±0.02 | 0.50±0.04 | 0.68±0.04 |
|  |  |  |  |  |  |  |
|  | Non-evolved | NOEV.1 | 0.97±0.06 | 0.51±0.04 | 0.47±0.04 | 0.75±0.02 |
|  |  | NOEV.2 | 1.08±0.10 | 0.50±0.04 | 0.43±0.04 | 0.72±0.03 |
|  |  | NOEV.3 | 1.04±0.07 | 0.41±0.06 | 0.50±0.02 | 0.71±0.02 |
|  |  | NOEV.4 | 0.89±0.08 | 0.50±0.05 | 0.40±0.04 | 0.64±0.04 |

^1^ Accumulation of virus RNA (μg/g fresh weight) estimated for 1:1 mix of inoculated and systemically infected leaves.

^2^ Effect of CMV infection on rosette weight (*RW*) and inflorescence weight (*IW*) estimated as Trait*_i_*/Trait*_m_*, where *i* and *m* denote infected and mock-inoculated plants, respectively.

^3^ Virulence estimated as one minus the ratio of seed weight in infected *vs.* mock-inoculated plants: 1-(*SW*_i_*/SW*_m_).

Values are mean±standard error of 10 replicates.

**Table S2.** *Continued*…

| **Strain** | **Transmission**  **Mode** | **Lineage** | **Virus**  **Accumulation^1^** | **Vegetative growth^2^:**  ***RW_i_/RW_m_*** | **Reproductive**  **growth^2^:**  ***IW_i_/IW_m_*** | **Virulence:**  **1-*(SW_i_/SW_m_)*^3^** |
| --- | --- | --- | --- | --- | --- | --- |
|  |  |  |  |  |  |  |
| LS-CMV | Vertical | LS.1 | 9.06±0.34 | 0.40±0.10 | 0.54±0.04 | 0.59±0.07 |
|  |  | LS.2 | 9.04±0.23 | 0.53±0.07 | 0.55±0.04 | 0.64±0.04 |
|  |  | LS.3 | 8.06±0.20 | 0.56±0.07 | 0.51±0.03 | 0.54±0.04 |
|  |  | LS.4 | 6.97±0.25 | 0.57±0.07 | 0.48±0.04 | 0.59±0.03 |
|  |  | LS.5 | 8.25±0.29 | 0.41±0.08 | 0.45±0.04 | 0.62±0.03 |
|  |  |  |  |  |  |  |
|  | Horizontal | LS.1 | 11.24±0.03 | 0.29±0.04 | 0.41±0.02 | 0.77±0.03 |
|  |  | LS.2 | 11.30±0.01 | 0.28±0.02 | 0.40±0.03 | 0.78±0.02 |
|  |  | LS.3 | 13.99±0.23 | 0.30±0.04 | 0.39±0.04 | 0.69±0.05 |
|  |  | LS.4 | 11.27±0.01 | 0.31±0.04 | 0.38±0.04 | 0.73±0.03 |
|  |  | LS.5 | 11.29±0.02 | 0.26±0.05 | 0.31±0.04 | 0.78±0.03 |
|  |  |  |  |  |  |  |
|  | Alternate | LS.1 | 9.43±0.38 | 0.38±0.06 | 0.40±0.05 | 0.72±0.04 |
|  |  | LS.2 | 8.76±0.38 | 0.40±0.06 | 0.47±0.03 | 0.70±0.02 |
|  |  | LS.3 | 9.91±0.62 | 0.46±0.06 | 0.42±0.02 | 0.70±0.03 |
|  |  | LS.4 | 8.64±0.06 | 0.37±0.05 | 0.37±0.05 | 0.68±0.05 |
|  |  | LS.5 | 6.97±0.07 | 0.34±0.09 | 0.37±0.05 | 0.68±0.03 |
|  |  |  |  |  |  |  |
|  | Non-evolved | NOEV.1 | 17.46±3.42 | 0.23±0.04 | 0.37±0.05 | 0.75±0.03 |
|  |  | NOEV.2 | 20.72±1.74 | 0.26±0.04 | 0.34±0.07 | 0.77±0.03 |
|  |  | NOEV.3 | 20.71±0.94 | 0.27±0.04 | 0.34±0.04 | 0.76±0.04 |
|  |  | NOEV.4 | 19.85±1.70 | 0.23±0.05 | 0.38±0.02 | 0.77±0.01 |
|  |  | NOEV.5 | 20.35±1.03 | 0.20±0.02 | 0.38±0.02 | 0.76±0.03 |

^1^ Accumulation of virus RNA (μg/g fresh weight) estimated for 1:1 mix of inoculated and systemically infected leaves.

^2^ Effect of CMV infection on rosette weight (*RW*) and inflorescence weight (*IW*) estimated as Trait*_i_*/Trait*_m_*, where *i* and *m* denote infected and mock-inoculated plants, respectively.

^3^ Virulence estimated as one minus the ratio of seed weight in infected *vs.* mock-inoculated plants: 1-(*SW*_i_*/SW*_m_).

Values are mean±standard error of 10 replicates.
